# The Role of Prefrontal Cortex in Working Memory: A Mini Review

**DOI:** 10.3389/fnsys.2015.00173

**Published:** 2015-12-18

**Authors:** Antonio H. Lara, Jonathan D. Wallis

**Affiliations:** ^1^Department of Neuroscience, Columbia University Kolb Research AnnexNew York, NY, USA; ^2^Helen Wills Neuroscience Institute, University of California, BerkeleyBerkeley, CA, USA; ^3^Department of Psychology, University of California at BerkeleyBerkeley, CA, USA

**Keywords:** working memory, attention, executive function, prefrontal cortex, frontoparietal network

## Abstract

A prominent account of prefrontal cortex (PFC) function is that single neurons within the PFC maintain representations of task-relevant stimuli in working memory. Evidence for this view comes from studies in which subjects hold a stimulus across a delay lasting up to several seconds. Persistent elevated activity in the PFC has been observed in animal models as well as in humans performing these tasks. This persistent activity has been interpreted as evidence for the encoding of the stimulus itself in working memory. However, recent findings have posed a challenge to this notion. A number of recent studies have examined neural data from the PFC and posterior sensory areas, both at the single neuron level in primates, and at a larger scale in humans, and have failed to find encoding of stimulus information in the PFC during tasks with a substantial working memory component. Strong stimulus related information, however, was seen in posterior sensory areas. These results suggest that delay period activity in the PFC might be better understood not as a signature of memory storage *per se*, but as a top down signal that influences posterior sensory areas where the actual working memory representations are maintained.

## Introduction

A widely held view of prefrontal cortex (PFC) function is that it encodes task relevant information in working memory (Goldman-Rakic, 1987; Miller and Cohen, 2001; Baddeley, 2003). This account originates from decades of work that showed strong neural activity in PFC during the delay period of working memory tasks (Fuster and Alexander, 1971; Funahashi et al., 1993a; Wilson et al., 1993; Levy and Goldman-Rakic, 2000). This delay period activity has two key properties. First, it is specific to the stimulus being remembered, consistent with it containing information about the content of working memory. Second, it only encodes stimuli that are relevant to the task at hand: it is resistant to distractors (Miller et al., 1996; Sakai et al., 2002) and task irrelevant information is not encoded in working memory (Rainer et al., 1998). These properties of delay period activity have been observed at the single-neuron level in monkeys as well as on a larger scale in human imaging studies (Courtney et al., 1998; Zarahn et al., 1999; Curtis et al., 2004). In monkeys, single neurons recorded from PFC maintain stimulus information across the delay period, even when distracting stimuli are presented in the middle of the delay (Miller et al., 1996). The delay period activity is thought to reflect the stimulus currently in memory (Fuster, 1973; Funahashi et al., 1993a; Wilson et al., 1993; Procyk and Goldman-Rakic, 2006). In humans, multiple studies using various imaging techniques have also shown an increase in delay period activity in PFC. For example, using functional magnetic resonance imaging (fMRI) sustained activation was measured in the lateral PFC while subjects kept spatial locations in working memory across delays of several seconds (Courtney et al., 1998).

The necessity of PFC delay activity for working memory is demonstrated by studies showing that lesions to PFC produce strong deficits in working memory tasks both in monkeys (Fuster and Alexander, 1971; Bauer and Fuster, 1976; Funahashi et al., 1993b; Wilson et al., 1993; Levy and Goldman-Rakic, 2000) and humans (Müller et al., 2002; Tsuchida and Fellows, 2009; Voytek and Knight, 2010). In addition, disruption of delay period activity with microstimulation increases the rate of errors (Wegener et al., 2008). Furthermore, the longer the delay, the greater the error rate, consistent with a failure of working memory to retain stimulus information. These findings have formed the basis for the prevailing view of that PFC is the site where information about the stimulus to be remembered is stored in working memory (for a recent review, see D’Esposito and Postle, 2015). However, recently there has been a growing body of work that has cast doubt on this theory (Druzgal and D’Esposito, 2001; Curtis and D’Esposito, 2003; Postle et al., 2003; Ranganath et al., 2004; Sreenivasan et al., 2014a,b; Postle, 2015). In this mini-review, we will briefly discuss the evidence against the prevalent theory and review emerging evidence for an alternate proposal for the role of PFC in working memory.

## Is PFC the Site of Working Memory Storage?

Some of the first evidence that contradicted the view that PFC represents stimulus information in working memory came from neuroimaging studies in humans. Researchers showed that delay period activity in PFC did not encode information specific to the stimulus being held in working memory (Curtis and D’Esposito, 2003; Riggall and Postle, 2012), while the converse was true for posterior sensory areas (Ester et al., 2009; Harrison and Tong, 2009; Serences et al., 2009; Emrich et al., 2013). These findings are important because they confirm that PFC is active during the delay period. However, they also suggest that PFC does not contain information about the stimulus, as would be expected if PFC were the site of working memory storage. In addition to evidence from imaging studies, it has been reported that lesions of PFC do not always impair working memory storage. Patients with large lesions localized to the lateral PFC showed no deficits on tests of verbal and memory span or delayed recognition (D’Esposito and Postle, 1999). A similar result was found in monkeys with lesions of the ventral PFC (Rushworth et al., 1997).

In trying to reconcile these discrepant findings, Curtis and D’Esposito (2003) proposed an alternate role for delay period activity in PFC: “*the [dorsal lateral] PFC does not store representations of past sensory events or future responses. Instead, its activation is an extra-mnemonic source of top-down biasing control over posterior regions that actually store the representations.*” A similar proposal was put forward by Postle (2006), based on similar line of evidence from lesion, imaging and electrophysiology studies. In his influential review Postle argued that “*the retention of information in working memory is associated with sustained activity in the same brain regions that are responsible for the representation of that information in non-working memory situations*”; this implies “*that the PFC is not a substrate for the storage of information in working memory.*” (Postle, 2006) Instead, according to Postle, the contribution of PFC to working memory could be any of the control processes (e.g., attentional selection, flexible control, etc.) that are also required when performing a working memory task.

Until recently, however, there was little electrophysiological evidence to support these views. In an early study, Lebedev et al. (2004) trained monkeys to maintain one spatial location in working memory while they also attended to a different location that would provide the go cue for making a saccade to the remembered location. They found two populations of neurons in PFC: one population encoded the location where the monkeys were attending while the other population encoded the spatial location in working memory (Lebedev et al., 2004). This was one of the first demonstrations that PFC neurons can play a different role in a working memory task that is not strictly maintenance *per se*. Additional evidence for an alternate role PFC in working memory tasks comes from recent work in which researchers used multivariate pattern analysis of neuronal data recorded during performance of a delayed paired-associate task (Stokes et al., 2013). During initial stimulus presentation, PFC population activity encoded information related to the stimulus, yet this information did not persist into the memory period. During subsequent stimulus presentations, PFC population activity first encoded the physical properties of the new stimulus and shortly thereafter it switched to code whether it was a target or a distractor. Thus, PFC does not maintain stimulus information in working memory *per se*, yet it has access to that information and can reliably encode whether subsequent stimuli are targets or distractors.

Our own work has demonstrated further evidence that PFC is not necessarily involved in maintaining stimulus information in working memory (Lara and Wallis, 2014). We trained monkeys to perform a multi-item working memory task in which they had to remember the color of one or two colored squares. We used a large set of colors and the discriminations could be very difficult, often involving subtle changes in the shade of color. The difficulty of the discriminations required that monkeys maintain a very precise representation of the sample colors in working memory in order to successfully perform the task. Despite the difficulty of the task, monkeys could perform the task significantly above chance level. Surprisingly, however, we found that the overwhelming majority of PFC neurons failed to encode the color of the stimuli in working memory. Instead, the strongest signals reflected the passage of time and the spatial location of the stimuli. Both of these signals could play an important role in organizing behavior towards the performance of the task, but they do not reflect the contents of working memory.

On further analysis, we found that when monkeys had to maintain two colors in working memory, they tended to make small eye movements (microsaccades) to one or other of the items. These microsaccades had behavioral consequences and appeared to reflect covert attention. If the animals covertly attended an item, it was stored with a more precise representation in working memory. The animals appeared to be shifting their attention between the items in order to cope with the increased task difficulty. In this situation, neural activity strongly reflected the locus of covert attention. These results directly support the ideas put forward by Postle (2006). Even though the key requirement of the task was to maintain color information in working memory, there was very little evidence that PFC neurons encoded color. But this did not mean that PFC was uninvolved in the task. Instead PFC neurons encoded attentional control signals that helped improve the animals’ performance.

In addition to the emerging neurophysiological evidence discussed above, a recent lesion study bolsters the case against the prevalent view of PFC function in working memory. Pasternak et al. (2015) trained monkeys to perform a delayed-match-to-sample task using random dot stimuli of varying motion coherence. Researchers found that lesions of the lateral PFC produced moderate deficits in the monkeys’ ability to remember the direction of motion of stimuli presented in the contralesional side. However, this deficit did not depend on the specific features of the stimuli that led to the remembered direction of motion (e.g., motion coherence), indicating that PFC was not involved in coding the specifics of the motion stimulus. Furthermore, deficits were much more pronounced when the sample and test stimuli appeared in different locations compared to when they appeared in the same location. Thus, PFC lesions seemed to disrupt the ability of the monkeys to rapidly shift their attention at the time of the test. Pasternak and colleagues interpreted these results as evidence that PFC plays a role in attending to stimuli and accessing motion information stored in other areas.

## Sensory Cortices Play a Critical Role in Working Memory

If PFC is not responsible for storing information in working memory, then it is important to identify those brain areas that are responsible for this process. There is strong evidence from electrophysiological and functional imaging studies that sensory cortices play a crucial role (Pasternak and Greenlee, 2005). A large number of electrophysiology studies have examined single neuron activity in most sensory cortices including visual (Miller et al., 1993; Motter, 1994), auditory (Gottlieb et al., 1989), and even gustatory cortex (Lara et al., 2009). For example, working memory related activity has been reported in area V4 in a task where monkeys had to remember the color or luminance of a stimulus (Motter, 1994). A number of functional imaging studies have also reported working memory activity in sensory cortices. For example, in a study in which participants had to remember the orientation of a grating (Ester et al., 2009; Harrison and Tong, 2009; Serences et al., 2009; Emrich et al., 2013), orientation specific activation patterns were observed in the pooled activity of early visual areas V1–V4.

If posterior sensory areas are responsible for keeping information in working memory while PFC plays a role in attending to or selecting this information, then there must be a mechanism by which PFC and posterior sensory areas can interact. This assumption is not outlandish since it is known that PFC has reciprocal connections with nearly all sensory cortices (Pandya and Barnes, 1987). What is the nature of the interaction? One possibility is that PFC and posterior areas share information through long-range coupling of ongoing oscillatory activity present in both areas (Engel et al., 2001; Fries, 2009; Canolty and Knight, 2010). Indeed, there is a large body of work both in monkeys and in humans that has revealed an important role of oscillatory activity during working memory tasks (Vogel and Machizawa, 2004; McCollough et al., 2007; Ikkai et al., 2010; Johnson et al., 2011; Myers et al., 2014). For example, in monkeys, strong oscillatory activity in the local field potential (LFP) has been seen in lateral intra-parietal cortex during the performance of a delayed saccade task (Pesaran et al., 2002), and in V4 of monkeys performing a delayed match to sample task (Tallon-Baudry et al., 2004; Lee et al., 2005). There have also been reports of strong oscillatory activity in the LFP of PFC during the delay period (Siegel et al., 2009; Lara and Wallis, 2014) of delayed match to sample tasks.

In humans, extensive work using electroencephalography (EEG), electrocorticography (ECoG) and magneto-encephalography (MEG) has revealed increased ongoing oscillatory activity during working memory tasks both in frontal and posterior areas (for a review, see Roux and Uhlhaas, 2014). In a recent study, participants were asked to remember the spatial locations of either three red discs, three red discs while ignoring three blue discs or six red discs (Roux et al., 2012). In all conditions there was increased oscillatory MEG activity in the alpha and gamma frequency bands. In PFC, activity in the gamma-band (which is thought to reflect local processing; von Stein and Sarnthein, 2000) predicted the amount of task relevant information in working memory. A linear classifier using gamma-band activity from PFC could successfully classify trials with three targets and three distractors in the same category as trials with only three discs and not as six disc trials. Thus, the classifier correctly ignored the task irrelevant discs. In contrast, gamma-band activity in the inferior parietal lobule also reflected spatial information during the delay period, but the classifier failed to identify distractor trials as three item trials. Thus, it appears that while gamma-band activity in both PFC and parietal cortex reflects the stimuli currently in memory, only in PFC is the information discriminated as either task relevant or task irrelevant. A similar result was seen in monkeys where ventral intraparietal cortex population activity robustly encoded the number of target stimuli in a delayed-match-to-numerosity task even in the face of distractors (Jacob and Nieder, 2014). In contrast, PFC population briefly encoded distractors, but target numerosity information was quickly restored and the strength of the restored information predicted correct performance in a trial. Again, this suggests that PFC is not simply involved in the storage of information, but reflects control processes such as monitoring and selection.

## Interactions between PFC and Sensory Cortex

In order to fully understand the nature of the interaction between PFC and posterior sensory cortices, it is important to measure neural activity in both areas simultaneously. A number of recent studies have managed to do this during the performance of working memory tasks. A recent study examined the interaction between V4 and lateral PFC using simultaneous LFP and single neuron recordings in monkeys performing a visual working memory task (Liebe et al., 2012). In this study, researchers found that the theta-band phase locking value, a measure that quantifies the amount of synchrony between theta oscillations in V4 and PFC, was significantly enhanced during the delay period. The phase of PFC oscillations led V4 by about 15 ms, which suggests that the observed coupling is asymmetric and sufficiently fast to support functional interactions between the two areas. Indeed, when they looked at the timing of the spikes from each area, they found that during the delay, spike times were reliably locked to the phase of the ongoing delta-band oscillations in the more distant area (i.e., PFC spikes were phase locked to V4 delta-band LFP and vice versa). Importantly, these effects were stronger in trials in which monkeys successfully maintained information in working memory, and weaker in trials in which monkeys failed to remember the stimulus. These results suggest that synchronous activity in PFC and V4 could provide a mechanism through which information is shared between these two distant areas during working memory maintenance.

A similar flow of information was recently observed between PFC and posterior parietal cortex (Salazar et al., 2012). In this study, researchers made simultaneous spike and LFP recordings from PFC and posterior parietal cortex while monkeys performed a spatial delayed match to sample task. They calculated a coherence selectivity index designed to measure how much mutual information about the memorized stimulus there is between PFC and parietal electrodes. An increase in mutual information about sample identity and location was observed during the delay period. Furthermore, Weiner-Granger Causality showed that the flow of information was primarily from parietal cortex to PFC. These results are consistent with the idea that the storage of information is taking place in sensory cortex and PFC can access that information through synchronization of oscillatory field potentials. A similar phenomenon was reported in a recent study where researchers simultaneously recorded neural activity from lateral PFC and lower level visual areas MT and MST while monkeys performed a delayed match to sample task (Mendoza-Halliday et al., 2014). During the delay period, increased selective spiking activity was seen in MST and lateral PFC but not in MT. This sustained spiking could conceivably reflect the maintenance of stimulus information in working memory in both brain areas. However, an alternative possibility is that MST maintains a strong representation of the stimulus in working memory, which is then read out and integrated with other higher order signals by PFC. The behavioral task does not permit these two possibilities to be distinguished. However, even though there was no increase in spiking activity in MT during the delay period, stimulus information was present in the LFP amplitude from this area. Moreover, there was increased synchrony between low frequency LFP oscillations in MT and lateral PFC spikes, consistent with a top-down interaction between the PFC and early sensory neurons during the maintenance period.

Long-range synchronization of oscillatory field potentials is likely not the whole story. There is also the possibility of a more direct interaction via cortico-cortical synaptic connections between PFC and posterior sensory neurons (Petrides and Pandya, 1984). In a recent study, Crowe et al. (2013) recorded single neuron activity simultaneously form PFC and posterior parietal cortex neurons while monkeys were engaged in a categorization task. Both PFC and parietal neurons have been shown to play an important role in categorization tasks of this kind (Freedman et al., 2001; Miller et al., 2002; Wallis and Miller, 2003; Freedman and Assad, 2006; Ferrera et al., 2009; Swaminathan and Freedman, 2012). They found that the pattern of firing in PFC was strongly correlated with the pattern of firing in posterior parietal cortex at different time lags. Crucially, there was significantly stronger correlation between the pattern of PFC activity at one time and PPC activity at a later time, compared to the opposite direction. These results reflect selective top-down transmission of information from prefrontal to parietal neurons via a mechanism that does not necessarily involve synchronization of ongoing oscillatory activity. Although these results were found in a categorization task, a similar phenomenon could be at play during working memory. Furthermore, the exact direction of the interaction may depend on the precise cognitive process being performed. For example, accessing sensory information may involve information flowing from parietal cortex to PFC (“bottom-up”), while selective attention and filtering may involve information flowing in the reverse direction (“top-down”). Recent studies of sensorimotor processing have shown such bidirectional interactions within the fronto-parietal network (Siegel et al., 2015).

One potential challenge to the view outlined in this review is the recent work by Ester et al. (2015). They required subjects to maintain very precise representations of oriented gratings in working memory, and showed that orientation information could be decoded from the BOLD signal in localized frontoparietal subregions. However, an important caveat in interpreting these kinds of results is that information can be decoded even when neurons are not representing that information. For example, orientation information can be decoded from the retina in principle even though no individual neuron is representing orientation information. In an analogous way, it is possible that orientation information could be decoded from the pattern of activity in PFC neurons responsible for activating the correct representation in posterior sensory cortex even though individual PFC neurons are not tuned for this information in their firing rate. On the other hand, if PFC neurons responsible for precise sensory representations are localized to small subregions it is possible that these representations are missed by standard sampling methods used in single-unit neurophysiology studies. This possibility could be excluded by recording neural activity at multiple scales, such as combining ECoG and single unit methods (Lewis et al., 2015).

## Conclusion

In recent years there has been steady stream of work that has challenged the widely held view that PFC stores task relevant information in working memory. Early evidence against this view came mainly from fMRI studies in humans and it culminated in the alternate view, most clearly enunciated by Postle (2006), that sensory information is maintained in working memory by the same sensory neurons that represent that information when it is present in the sensory environment. The role of PFC is not to store information in working memory, but rather to actively focus attention on the relevant sensory representation, select information and perform executive functions that are necessary to control the cognitive processing of the information (Postle, 2006). There is growing neurophysiological and lesion evidence in support of this view.

More work is needed to shed light on the precise nature of the interaction between PFC and sensory areas during working memory. The use of modern large-scale recording methods (Kipke et al., 2008) and analysis techniques (Cunningham and Yu, 2014) has the potential to allow the tracing of the flow of information from sensory areas to PFC and back again during working memory tasks. Equally as important, however, is to lay in place a theoretical framework that will allow the interpretation of this data. One promising idea is to try and understand how neuronal activity is related to the internal state of the brain above and beyond any coding for external factors. This approach forms the basis of the dynamical-systems framework, which has recently been adopted to understand the neural mechanisms underlying motor control (Shenoy et al., 2013). Given that executive processes like working memory and attention are, by their very nature, internal, dynamical processes, using a dynamical-systems approach in their study has the potential to shed light on how the brain internally generates (i.e., without relying on external inputs) the patterns of activity that are required for such a complex repertoire of executive abilities.

## Funding

This work was supported by NIMH grant R01-MH097990 and NIDA grant R01-DA19028 to JDW.

## Conflict of Interest Statement

The authors declare that the research was conducted in the absence of any commercial or financial relationships that could be construed as a potential conflict of interest.
